# Eosinophilic esophagitis improved by the discontinuation of sublingual immunotherapy for aeroallergens: a case report

**DOI:** 10.3389/fped.2024.1379162

**Published:** 2024-03-19

**Authors:** Alice Monzani, Marta Cerutti, Sara Curto, Sara Lovaste, Marta Coppola, Francesca Mercalli, Silvia Saettone, Ivana Rabbone

**Affiliations:** ^1^ Division of Pediatric, Department of Health Sciences, University of Piemonte Orientale, Novara, Italy; ^2^ Unit of Pathology, Maggiore Della Carità University Hospital, Novara, Italy; ^3^ Gastroenterology Unit, Maggiore Della Carità University Hospital, Novara, Italy

**Keywords:** eosinophilic esophagitis, environmental aeroallergens, desensitization, sublingual immunotherapy, case report

## Abstract

**Introduction:**

Eosinophilic esophagitis (EoE) is a chronic, immune-mediated inflammation of the esophagus, characterized by symptoms related to esophageal dysfunction, resulting from severe eosinophilic infiltration of the esophageal mucosa. It is common in atopic subjects and food antigens have been identified as the most common triggers. However, a seasonal variation in EoE prevalence, correlated with air pollen levels, is reported, suggesting that also aeroallergens may play a role. Little is known about the interplay between EoE and concomitant atopy treatment for aeroallergens.

**Case presentation:**

We describe the case of an 11-year-old boy who presented dysphagia, vomiting, drooling, and chest pain while eating meat, developed 15 months after receiving sublingual immunotherapy (SLIT) for Alternaria (SUBLIVAC®). He underwent esophagogastroduodenoscopy (EGD) revealing severe eosinophilic predominant inflammation (100 eos/HPF), consistent with the diagnosis of EoE, not improving at the EGDs performed after both omeprazole and topical corticosteroids treatment, despite symptom improvement. Afterward, immunotherapy was switched from sublingual to injective form. At the EGD performed 1 month later, macroscopic examination of the esophageal mucosa was normal and eosinophilic infiltration was significantly decreased (5–10 eos/HPF).

**Conclusions:**

SLIT may induce EoE by chronic antigenic exposure of oral mucosa in patients with a robust allergic susceptibility: while attenuating the IgE-mediated immune reactions, the progressive contact with the causative allergen might induce a chronic stimulation of the immune system with the consequent activation of tissue eosinophils. Our data suggest monitoring patients receiving SLIT for EoE symptoms and to discontinue SLIT on their earlier appearance, possibly as a first-line treatment.

## Introduction

Eosinophilic esophagitis (EoE) is a chronic, immune-mediated and allergen-triggered inflammation of the esophagus, clinically characterized by symptoms related to esophageal dysfunction, resulting from severe eosinophilic infiltration of the esophageal mucosa (1). It is the leading cause of dysphagia and food impaction in children and young adults, with an estimated incidence in the pediatric population ranging from 0.7 to 10/100,000 per person-year, and a prevalence of 0.2–43/100,000 (1).

Patients with EoE usually suffer from concomitant atopic disorders including rhinitis, asthma, and eczema. Moreover, IgE-mediated food allergies are common in EoE patients. The risk of developing EoE in patients undergoing oral immunotherapy (OIT) for desensitization from IgE-mediated food allergy is reported to be 2.7%, mainly in children undergoing peanut, milk, and egg OIT (2). Recognizing the central role of chronic antigen exposure in the pathogenesis of EoE emphasizes the importance of accurately identifying all potential triggers for each patient. Although the majority of patients respond to food elimination diets, the lack of an adequate response to dietary changes in a portion of EoE patients implies that antigens other than food, such as aeroallergens, are capable of triggering EoE (3). Some authors have found a seasonal variation in the prevalence of EoE, correlated with air pollen levels, which confirms that aeroallergens may play a role in this disorder (4). It is reasonable that the exposure of oral mucosa to an aeroallergen, as it happens in sublingual immunotherapy (SLIT), would elicit an eosinophilic response at the upper gastrointestinal tract level, possibly triggering EoE in predisposed subjects. Up to now, there are few reports of patients -mainly adults- who developed EoE after being treated with SLIT against pollen and mites that remitted with the withdrawal of immunotherapy (5–9). We describe the case of a young boy, diagnosed with EoE while receiving SLIT for Alternaria, showing endoscopic improvement only with the SLIT discontinuation, after the failure of other therapeutic approaches.

## Case description

An 11-year-old boy, with a past medical history of asthma and allergic oculorhinitis, performed skin prick tests which showed a positive reaction to grass mix (Dactylis glomerata, Festuca elatior, Lolium multiflorum, Phleum pratense, Poa pratensis) (4 mm diameter), bermuda grass (4 mm), ragweed (4 mm) and alternaria (6 mm). The specific IgE against molecular Alt a1 identified Alternaria as the main allergen (M229 84.90 kU/L). Therefore, treatment with a daily oral allergen extract of Alternaria by sublingual administration (SUBLIVAC® HAL Allergy—Leiden, The Netherlands) was initiated (drops placed under the tongue for 2 min and then swallowed).

**Figure 1:**
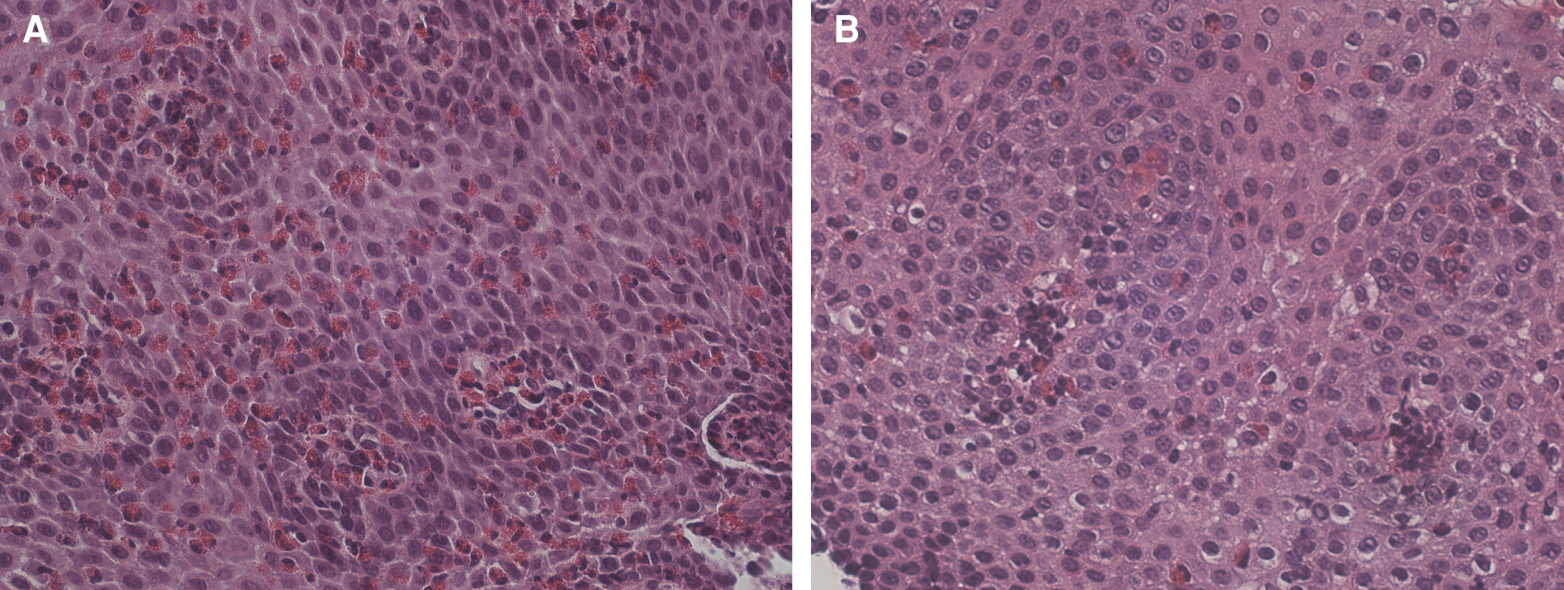
(**A**) Dense intraepithelial inﬁltration of eosinophils with formation of micro-abscesses. Dilated intracellular spaces are evident. HE 400×. (**B**) Slides after stopping sublingual immunotherapy reveal marked reduction of eosinophils and intercellular edema. HE 400×.

Fifteen months later, the patient was admitted to our pediatric emergency department for dysphagia, vomiting, drooling, and chest pain appeared while eating meat. His physical examination and vital signs were normal. The complete blood cell count showed mild peripheral eosinophilia (0.59 × 103 /μl). Upper gastrointestinal endoscopy revealed linear furrowing of the mucosa of the middle and proximal esophagus. Five esophageal biopsy specimens (2 from the distal third, 1 from the middle third, and 2 from the proximal third) were obtained. Histopathologic examination revealed severe eosinophilic predominant inflammation with infiltration of about 100 eos/HPF in nearly all biopsies, consistent with the diagnosis of EoE (Figure 1A).

**Figure 2:**
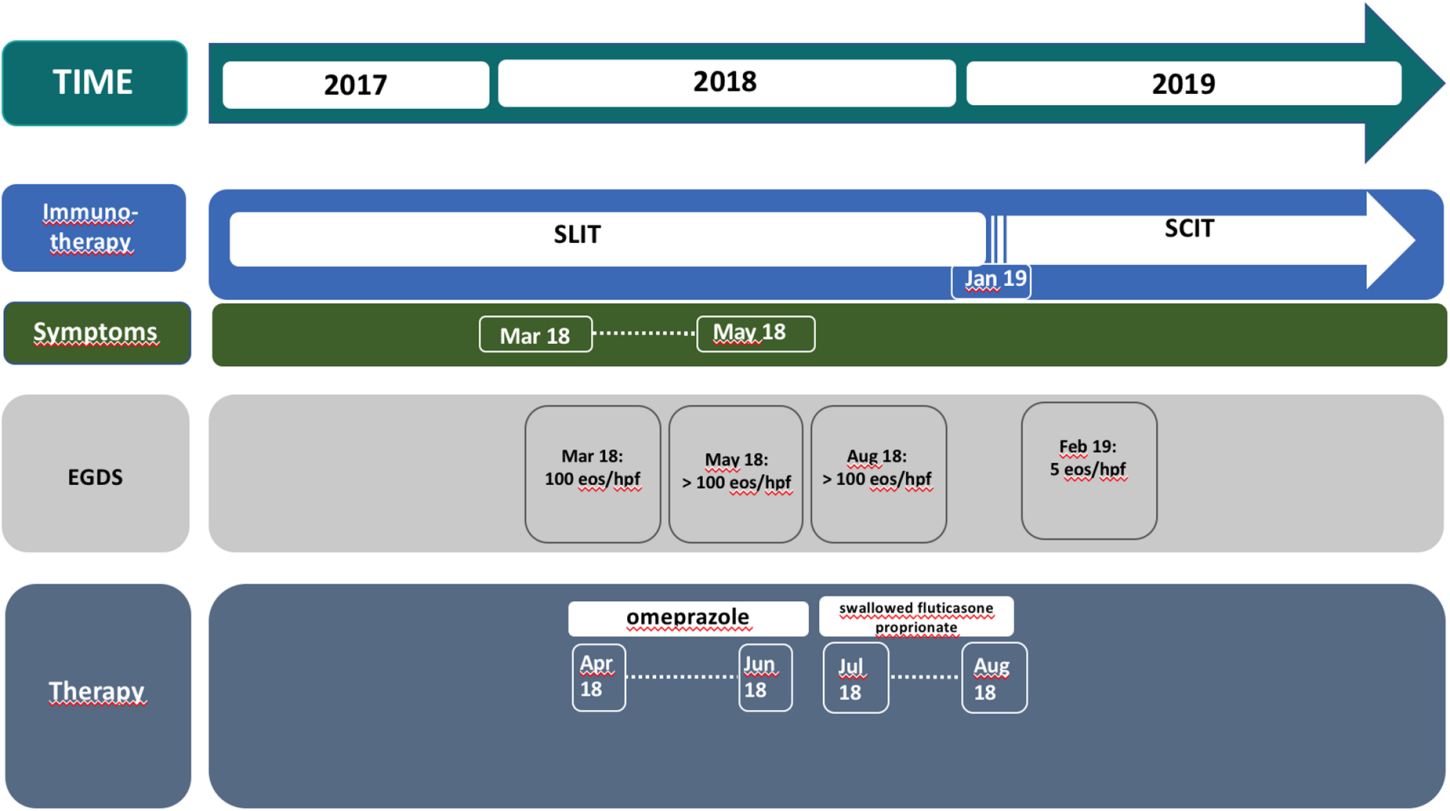
Timeline from the episode of care.

Therapy with omeprazole 1 mg/kg/day was prescribed for 8 weeks, with symptomatic relief. After 7 weeks of treatment, the patient underwent a second upper gastrointestinal endoscopy that revealed the persistence of linear furrowing and eosinophilic infiltration (>100 eos/HPF) (Figure 2). Thus, a new treatment with topical corticosteroids was started (swallowed Fluticasone Propionate 250 μg, 1 puff/4 times/day). Eight weeks later, upper gastrointestinal endoscopy showed an unchanged macroscopic aspect and the persistence of eosinophilic infiltration (>100 eos/HPF) (Figure 2). In the empirical attempt to address an elimination diet, trying to avoid an extensive elimination approach, which would be hardly accepted by the patient, we performed PATCH tests which were negative for the most common food allergens (milk, egg, soy, cereals, fish, peanuts). Before starting an extensive elimination diet, we decided to try to discontinue SLIT and to switch to injective subcutaneous immunotherapy (SCIT) for Alternaria. One month after SLIT discontinuation, upper gastrointestinal endoscopy showed a normal esophageal mucosa with a diffuse decrease of eosinophilic infiltration (5–10 eos/HPF), and only one specimen still consistent with EoE diagnosis (21 eos/HPF) (Figure 1B). During a follow-up of 15 months, the patient remained free of any esophageal symptoms and his family refused further endoscopic control (Figure 2).

## Discussion and conclusions

Little is known about the interplay between eosinophilic esophagitis and atopy treatment. Only three previous reports exist about the association between EoE and SLIT for aeroallergens in children (7, 8). Benè et al. (7) reported a 10-year-old girl with asthma and allergic rhinitis who developed EoE 6 weeks after starting SLIT with standardized Dermatophagoides pteronyssinus and Dermatophagoides farinae extracts. Esophageal biopsy showed more than 100 eosinophils/HPF, with no response to proton pump inhibitors (PPI). Endoscopy 3 months after SLIT discontinuation revealed complete resolution of EoE. Rokosz et al. (8) also reported a 9-year-old autistic enteral feeding tube-dependent boy who developed EoE after initiating SLIT for grass (bermuda and rye) pollen, tree (eastern cottonwood, mesquite, orange, and olive) pollen, and dust mites. Esophageal tissue biopsy showed more than 57 eosinophils/HPF, with no response to PPI, elemental diet, and local corticosteroids. Esophageal eosinophilia was resolved in the follow-up endoscopy 1 year after SLIT discontinuation.

More recently, Wells et al. (9) described a case of a 10-year-old boy with severe grass-pollen related allergic rhinitis and a quiescent EoE who developed a new disease flare after only 1 week of grass pollen SLIT.

In our patient, EoE symptoms were reported after a long time of SLIT maintenance (15 months). He underwent serial endoscopies testifying treatment failure with PPI and topical corticosteroids in endoscopic healing (reported in the literature in 30%–70% and 29%–49% of treated patients, respectively) (10–12), and conversely demonstrating the efficacy of SLIT discontinuation. The repeated endoscopies after each therapeutic line suggested for the first time in a pediatric patient that SLIT-associated EoE may be a reversible process after SLIT discontinuation, non-responsive to other established EoE treatments.

As nowadays SLIT is widely used in the treatment of allergies caused by aeroallergens, it could be useful to monitor patients receiving SLIT for the appearance of symptoms suggestive of EoE (abdominal pain, reflux, dysphagia, drooling, food impaction, or chest pain). Nonetheless, gastrointestinal symptoms like abdominal pain, diarrhea, nausea, and vomiting are reported as possible adverse effects of SLIT, also in children (13). In the absence of endoscopic examination, it is impossible to say if they may be attributable to EoE. Therefore, it is likely that the actual prevalence of EoE in children receiving SLIT is underestimated.

The best timing to perform an endoscopic examination in the presence of gastrointestinal symptoms should be evaluated with the strict collaboration between pediatric allergologists and gastroenterologists, aimed to identify EoE-suggestive symptoms, based on validated questionnaires (14), administered at baseline and at fixed time-points during SLIT.

A timely diagnosis of SLIT-related EoE would prompt to SLIT discontinuation, possibly as the first therapeutic approach, given the lack of efficacy of other treatments, as seen in our patients and in previous reports. However, further studies are needed to assess the real efficacy of SLIT discontinuation as the first therapeutic line in EoE.

Furthermore, as only a few patients receiving SLIT develop EoE, it would be extremely useful to identify possible predictive factors (anamnestic or laboratory variables) associated with the risk of developing EoE. In this perspective, there have been multiple reviews demonstrating distinct seasonal variability in EoE diagnosis (15) and that aeroallergens may affect esophageal eosinophil counts and determine a flare of symptoms in certain atopic EoE patients (16). In our case, Alternaria is a perennial antigen, therefore a clear seasonality could not be identified. It might be hypothesized that patients reporting esophageal symptoms during seasons of higher aeroallergen exposure could be those at higher risk for developing SLIT-induced EoE. Further studies are needed to best determine who is most at risk for the development of SLIT-induced EoE.

## Data Availability

The raw data supporting the conclusions of this article will be made available by the authors, without undue reservation.
